# Abundance of antibiotic resistance genes in bacteria and bacteriophages isolated from wastewater in Shiraz 

**DOI:** 10.22099/mbrc.2021.39468.1584

**Published:** 2021-06

**Authors:** Sahar Zare, Abdollah Derakhshandeh, Ali Mohammadi, Masoud Noshadi

**Affiliations:** 1 Department of Pathobiology, School of Veterinary Medicine, Shiraz University, Shiraz, Iran; 2 Department of Water Science and Engineering, School of Agriculture, Shiraz University, Shiraz, Iran

**Keywords:** antibiotic resistance gene, Bacteria, Phage, β-lactamases, Sulfonamides

## Abstract

Generally, the high widespread presence of antimicrobial resistance, and the next freeing into aquatic environments which provide a situation for transmission of these genes in water is because of the abuse of the antimicrobial drugs in both medicine and veterinary medicine. In aquatic environment, bacteriophages could have an important role in sharing antimicrobial resistance genes. The purpose of this study was to assess three important antibiotic resistance genes including two β-lactamases (blaTEM, blaSHV) and sul1 gene, referring to resistance to sulfonamides, in both bacteria and phage DNA fractions of wastewater samples, Shiraz, Iran, using polymerase chain reaction. The prevalence of those genes was extremely high and equal to 100% in bacterial DNA, while these rates were lower in phage DNA fractions as 66.66%, 66.66% and 58.33% for blaTEM, blaSHV and sul1, respectively. In conclusion, detection of mentioned genes in bacterial and phage DNA fractions from ambient water is considerable, so the possibility of harbouring and transferring of antibiotic resistance genes by phages needs to be explored in the future. Also, available data is a reputable endorsement that wastewater is a hotspot for these kinds of genes to spread in the environment. Based on our knowledge, this is the first report of blaTEM and bla SHV and sul1 genes in bacterial and phage DNA fractions insulated from urban wastewater and environment in Iran.

## INTRODUCTION

Nowadays, one of the most significant concerns in the world is antibiotic resistance, which is caused by overuse of antibiotics and population growth [1]. Some kinds of antibiotic resistance genes have naturally occurred in bacteria due to different ways including natural or induced mutation, transduction, conjugation and transformation, and also it may transfer to other microorganisms through different ways such as mobile genetic elements [2, 3]. Many researches have centered on detection of antibiotic resistance genes in plasmids or transposons while few have investigated the possible portion of phage-mediated transmitted antibiotic resistance genes [4, 5]. Latter studies noted that horizontal transfer of genetic information via bacteriophages has higher prevalence than formerly thought, and the environment have a significant role in transmission of antibiotic resistance gene by phages [6, 7, 8].

Resistant bacteria are common consequences of using antibiotics. The ability of resistance to drugs in bacteria is unlimited and this originates a global problem [9]. The majority of studies on antibiotic resistance genes based on arable bacteria known as opportunists or pathogens such as *Enterobacteriaceae *or the genera *Acinetobacter*, *Aeromonas,* *Burkholderia*, *Pseudomonas*, *Enterococcus*, *Staphylococcus* [10].

Raw wastewater is known as one of the richest habitats for antibiotic resistance genes such as resistance to tetracyclines, sulfonamides, aminoglycosides and -lactames (e.g. *tet*, *aac*, *dfr*, *sul*, *arm* and class A -lactamases) [11]. Lots of mentioned genes are plasmid encoded and several of them placed in the variable gene cassettes of integrons that provide an easy mobilization between bacteria [10, 12, 13].

Despite of minimal information on the phage-mediated antibiotic resistance gene transfer, several reports are available which mentioned that phages are potential particles transfer the resistance genes through generalized transduction, also some authors recommended that mobilization and confer resistance could happen by phages [14-18]. There are a few studies that have examined phage DNA fractions from sewage to investigate antimicrobial resistance genes [19, 20].

Among other antibiotics, -lactam antibiotics seem to be more clinically effective and less toxic, so they are extensively used as antimicrobials. The -lactam genes and penicillin-binding proteins, mostly encoded by chromosome or plasmid, established a resistance against -lactam antibiotics, but the allotment information of bacteriophages role is less [21]. One of the resistance mechanisms to these antibiotics is production of -lactamases which is common in Gram-negative bacilli. Among these bacteria, *Enterobacteriaceae *usually represents some -lactamase such as TEM, causing resistance to penicillin [22]. Most of -lactamases are obtained horizontal gene transmission and emerge significantly spread worldwide [21]. 

This study was managed to use raw, treated and untreated samples from wastewater in Shiraz, Iran, and assessed the prevalence of some antibiotic resistance gene in bacterial and bacteriophages DNA fractions by PCR. The following genes were evaluated: *bla*_TEM_, *bla*_SHV_, and *sul1*.

## MATERIALS AND METHODS

**Figure 1:**
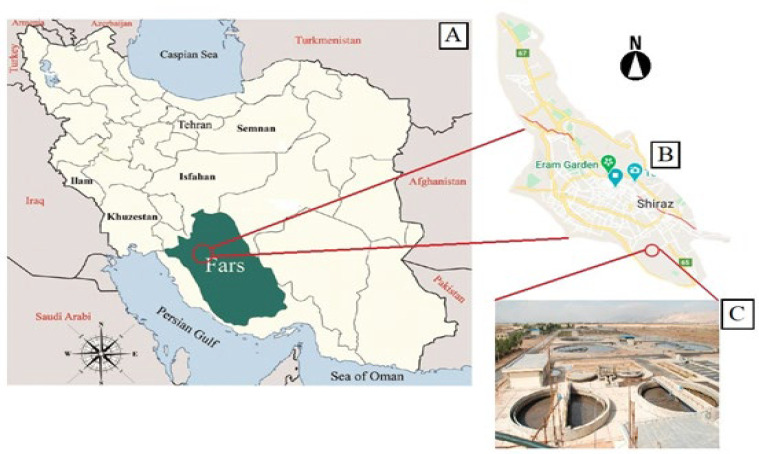
(A) Location of the Shiraz city in Iran. (B) Location map of the refinery in Shiraz. (C) Shiraz refinery

**Sampling** **:** Samples from Shiraz included wastewater in three locations at the refinery (incoming raw sewage, before treatment and after treatment) among 2015 and 2016. Samples were collected in sterile glass bottles, carried to the bacteriology lab at the School of Veterinary Medicine in Shiraz University beside ice about 3 hours of accumulation and processed for experiments (Fig. 1, Table 1). 

**Table 1:** Detail of samples

**Samples**	**Source**	**Season**
S1	incoming raw sewage	Spring
S2	Untreated sewage	Spring
S3	Treated sewage	Spring
S4	incoming raw sewage	Summer
S5	Untreated sewage	Summer
S6	Treated sewage	Summer
S7	incoming raw sewage	Autumn
S8	Untreated sewage	Autumn
S9	Treated sewage	Autumn
S10	incoming raw sewage	Winter
S11	Untreated sewage	Winter
S12	Treated sewage	Winter

**Purification of phage particles** **: **Purification was done according to Colomer-Lluch et al., 2011b with less adjustment [23]. Briefly, to partially purify viral particles 50 ml of each sewage samples was passed through low protein-binding 0.22-mm-pore-size membrane filters (Millipore, USA). Then the phages were 100-fold condensed using protein concentrators (Millipore, USA), following the manufacture protocol and final volume was diminished to 0.5 ml. The centrifugation time was assorted between10–50 min depending on the sample. Using double distilled sterile water, total volume adjusted to 2ml. To remove naked DNA in solution, DNase treatment was performed (100 units/ml of the viral concentrate) for 1 hour at 37°C. DNase activity was stopped by heating at 80°C for 10 min.

**Non-phage DNA control: **For this step, conventional PCR of eubacterial 16S ribosomal DNA (16SrDNA) was done on samples after DNase treatment, and before DNA extraction to ensure the absence of bacterial or non-encapsidated DNA.

**Purification of phage particle DNA** **: **DNA extraction from the phage particles started using proteinase K and phenol/chloroform (1:1) (v/v). The combination of phenol/chloroform/phage lysate centrifuged at 8000×rpm for 10 min. To precipitate the DNA from the supernatant, absolute ethanol and sodium acetate (3M) were used, then total volume was adjusted to 250 µl. At the end, purified DNA was eluted in a final volume of 50 µl and checked by agarose gel electrophoresis and NanoDrop ND-1000 spectrophotometer (Thermo Scientifics, USA) [24, 25].

**Bacterial DNA extraction:** According to Colomer-Lluch et al., 2014, 50 mL of each sewage sample was filtered by 0.45 μm membrane filters (Millipore, USA). Bacteria remained on the top of the membrane while phage particles were authorized to pass through the filters [24]. The filter which contains conserved bacteria was regained in 5 mL of LB broth (Merck, Germany), afterward the suspension centrifuged at 10,000×rpm for 10 min. Bacterial pellet was resuspended in 200 μL of LB broth, then the cells were heated to lyse at 95C for 10 min and immediately put in ice for 5 min. The supernatant was centrifuged at 12,000×rpm for 5 min, a final volume of 200 μL collected to fresh centrifuge tubes and kept at -20°C and DNA concentration was evaluated by a NanoDrop instrument (Thermo Scientifics, USA). The collected supernatant was preserved to use in amplification as a template source.

**PCR** **:** PCR assays for investigating the presence of the,* bla*_TEM_, *bla*_SHV_*,* and *sul 1* genes in bacterial and phage were performed using an MJ Mini thermal cycler (Bio-Rad, USA) and the primer pairs which used for PCR amplification were summed up in Table 2. All PCR reactions mixtures were the same and included 2.5 μL 10× PCR buffer, 0.75 μL dNTP (each at 0.2 mM), 0.2 μL *Taq* DNA polymerase (5 U/μL), 20 pmol of each primer, 0.75 μL 50 mM MgCl_2_ (CinnaGen, Tehran, Iran), and 3 μL of template DNA. PCR conditions for the detection of *bla*_TEM_ were as follows: 5 min at 95ºC, 15 sec at 94ºC, 30 cycles of 1 min at 63ºC, 1.3 min at 72ºC, and a final extension of 4 min at 72ºC. The amplification reactions for* bla*_SHV _and* sul 1*, were carried out as previously described while the annealing temperature was decreased to 60ºC and 58ºC respectively. PCR products were transferred to a 1% agarose gel, containing safe stain (CinnaGen, Tehran, Iran), electrophoresed and visualized under a UV transilluminator.

**Table 2:** Primers used for the amplification studies

**Target Gene**	**PCR**	**Sequence (5’ to 3’)**	**Amplimer(bp)**	**Reference**
16SrDNA	UP	AAGAGTTTGATCCTGGCTCAG	1503	[23]
	LP	TACGGCTACCTTGTTACGACTT		
blaTEM	UP	CTCACCCAGAAACGCTGGTG	569	[23]
	LP	ATCCGCCTCCATCCAGTCTA		
blaSHV	UP	GGCCGCGTAGGCATGATAGA	714	[61]
	LP	CCCGGCGATTTGCTGATTTC		
sul1	UP	ATGGTGACGGTGTTCGGCATTCTG	840	[62]
	LP	CTAGGCATGATCTAACCCTCGGTC		

**Electron microscopy** **:** The wastewater samples were refined by means of low protein-binding membrane filters (0.22-mm-pore-size) then concentrated up to 100-fold using protein condenser (Millipore, USA) conforming the instructions of manufacturer. Then, the phages suspension was deposited on copper grid with carbon-coated and stained with 2% KOH phosphotungstic acid (pH 7.2) for 5.0 min. At the end, the samples were examined with a Philips 906E transmission electron microscope operating at 80 kV.

**Statistical analysis** **:** The associations between sample groups of bacterial and viral samples regarding to the presence of antibiotic resistance genes were assessed by means of contingency Chi-squares ($\chi^{2}$ test) performed with the SPSS ver. 16.0 (SPSS Inc., Chicago, IL, USA) software.

## RESULTS

**Figure 2:**
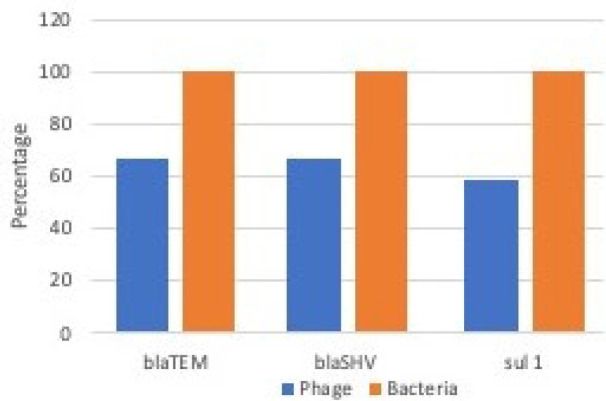
The presence rate of the three antibiotic resistance genes within the sample groups

**Figure 3:**
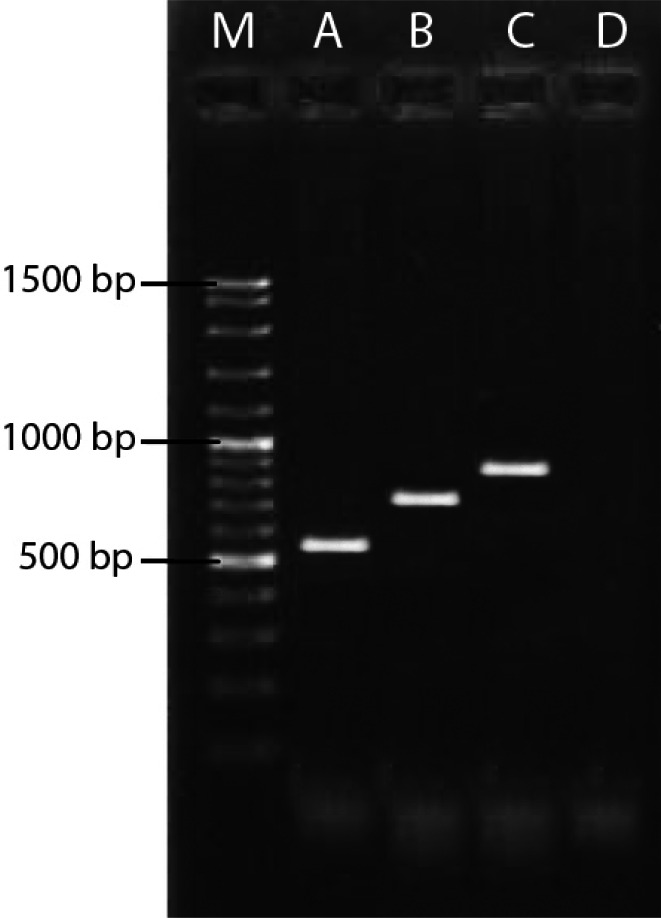
PCR patterns of three antibiotic resistance genes detected from bacterial DNA and phage DNA fractions isolated from wastewater. A:* bla*_TEM_, B:* bla *_SHV_, C:* sul1*, D: Negative Control and M: 100 bp DNA ladder

As specified before, to roll out that there was no DNA from a non-viral origin, a number of controls were tested to detect eubacterial 16S rDNA and three mentioned antibiotic resistance genes by conventional PCR and none of them showed the presence of these genes. According to the genotypic analysis by conventional PCR, all bacterial DNA showed the presence of *bla*_TEM_ and* bla*_SHV _and *sul1*, whereas this rate was 66.6%, 66.6% and 58.33% in phage DNA fractions respectively. Samples 3, 9 and 10 showed the absence of *bla*_TEM _and* bla*_SHV_. Also, *bla*_TEM _did not detect in sample 2 and and *bla*_SHV _did not detect in sample 5 (Figs. 2 and 3, Table 3). Accordingly, the ranking of abundance, as assessed by incidence of antibiotic resistance genes in bacterial and bacteriophages DNA fractions in Shiraz wastewater was *bla*_TEM_=*bla *_SHV_> *sul 1*.

**Table 3:** Distribution of antibiotics resistance genes among samples. ***N (%)*** presence of antibiotic resistance isolate in samples

**Gene**	**Bacterial DNA**	**Phage DNA fraction**	**Total**
blaTEM	12 (100)	8 (66.66)	20 (83.33)
bla SHV	12 (100)	8 (66.66)	20 (83.33)
Sul1	12 (100)	7 (58.33)	19 (79.16)

**Figure 4:**
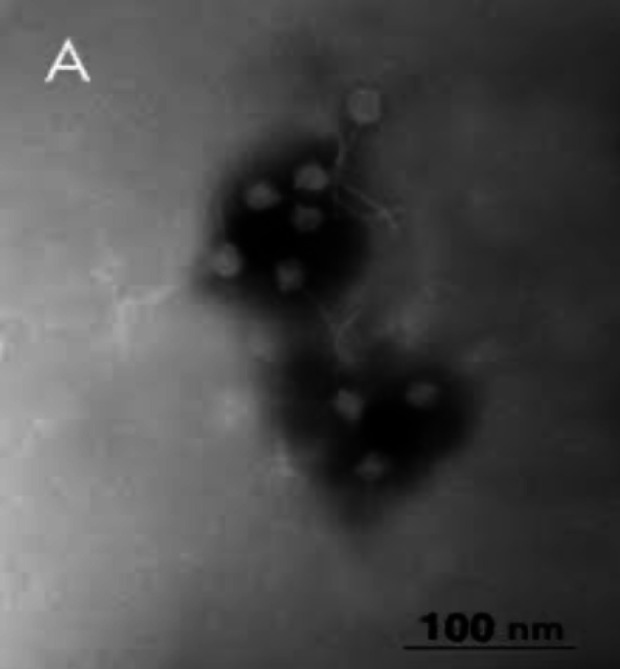
Electron micrographs of bacteriophages present in wastewater. A: Group of phages suspected *Myoviridae* morphology from sewage

Statistical analysis indicated that the bacterial and bacteriophage DNA fractions were not significantly related on existence of above antibiotic resistance genes. Bacteriophages that were presented in wastewater samples were directly observed by electron microscopy. Different morphological types of tailed phages were considered including contractile tail suspected *Myoviridae *morphology. Also, different size of capsid and tail were seen (Fig. 4).

## DISCUSSION

One of the very useful ways in fighting with infectious disease has been antibiotic therapy but the emersion and emergence of resistance between bacterial populations caused to fiddle away this achievement. Increasing antibiotic resistance could happen trough different and complex methods like mutation and horizontal gene transfer (HGT). An extensive variety of contaminant such as heavy metals and antibiotic resistant bacteria are endlessly subjected to the environment via wastewater depletion (treated and untreated), chattels activities, agronomy and manufacturing that may develop the emergence and transmission of antibiotic resistance [26- 28]. Additionally, different bacterial lineages could be found in different types of water, such as fresh water, fumigated water and raw wastewater. This is not deniable that these lineages can relocate relevant specifications, mostly those that can be transmitted by HGT, from polluted areas to the clean environments. However, human-associated microbiome was just a numerable group of water inhabitant bacteria but some of these lineages can indicate a connection amongst water habitats and human. Thus, those bacteria could be implicated in the direct or indirect transmission of properties, like antibiotic resistance [10].

Bacteriophages have a significant role in bacterial HGT and the following development of bacteria. Phages have some specifications include their redundancy and their versatile mechanisms in DNA-packaging which make them appropriate carrier for the attainment, conservation, and prevalence of antibiotic resistance genes [29]. Furthermore, researches showed that they endure treatment procedures in sewage from urban areas more than bacteria [30, 31]. This study searched the genes which are widely spread. All three studied genes have been reported from all over the world and spread in lots of population of bacteria such as ambient bacteria [32-35].

Antibiotic resistance genes have been noticed in phages and environmental bacterial strains from water bodies and urban wastewater in different studies [23, 25, 36-41]. In the present research antibiotic resistance genes have been observed in the environmental phage particles and bacteria. There was an obvious difference in the presence of the antibiotic resistance genes studied in the samples among bacterial DNA and DNA from the phage fractions, same as other studies, which described the presence of antibiotic resistance genes found in bacterial DNA was higher than that in bacteriophages [23-25]. *bla*_TEM_ and *bla *_SHV_ are more common antibiotic resistance genes and widely spread in the world [42-44]. In our study, all antibiotic resistance genes (*bla*_TEM_, *bla *_SHV_ and *sul 1*) were detected and all of them had 100% detection rate in bacterial DNA (Fig. 2, Table 3). 

The *bla*_TEM_ is a part of class A extended-spectrum β-lactamase (ESBLs) among the resistance encoding β-lactamase genes. The detection price of *bla*_TEM_ was acutely high in our research that was similar to other researches which worked on some of the environmental samples such as raw urban wastewater, sludge, river water, sediments and feces [37, 45-47]. The positive samples for *bla*_TEM_ found in phage DNA fractions were less than in bacterial DNA, as anticipated. The *bla*_TEM_ was detected in 100% of the bacterial DNA while, detection rate of this gene is 66.6% in bacteriophages DNA fractions these results are in agreement with other studies [24]. Moreover, 50% of phage DNA fractions that showed presence of *bla*_TEM_ were isolated from untreated and raw wastewater, which is in compromise with a previous study, where urban wastewater indicated a higher copy number of* bla*_TEM_ genes in compare with river water (Fig. 2, Table 3) [18, 23, 25].

The *bla*_SHV_ gene which is one of the most popular extended-spectrum β-lactamase (ESBL) genes, was present in all bacterial DNA and 66.6% of phage DNA fractions in this research. Fifty percent of positive phage DNA fractions were isolated from untreated and raw wastewater and of those phage DNA fractions that did not show the presence of this gene, only 16.66% (2 samples) were isolated from treated urban sewage. These results are in compromise with other studies [26, 47]. Regarding to the results the detection of a *bla *_SHV_ gene in bacterial and bacteriophage DNA is very high which is in accord with previous researches [26, 47]. In Addition, this rate of ESBL gens in the environment could consider an important threat to public health (Fig. 2, Table 3).

The *sul 1* is the most important antibiotic resistance genes against sulfonamides which is highly prevalence in the environment. In this study, *sul 1 *showed very high frequency in bacterial and phage samples isolated from sewage (100% of bacterial DNA and 58.33% of phage DNA) that is confirmed by other researches [18, 26, 34, 37, 45, 47, 48].

Generally, wastewater treatment process should cause a marked decrease in the sewage’s microorganism concentration before evacuation [49, 50]. As yet, a substantial number of resistance bacteria is detected in treated wastewater [51-53]. Furthermore, disinfection treatment may reduce the amounts of microorganisms but antimicrobial resistance bacteria and therefore antibiotic resistance genes in the total bacterial population could have ascending trend within sewage treatment [14, 54]. In addition, because the antibiotic resistance genes detected in the phage DNA fractions of the samples are surrounded by a protein made capsid, they are more persistent [30]. As previously mentioned, in this study there was not any significant changes in the present of antibiotic resistance genes in wastewater samples before and after treatment, same results were detected in a recent study too [55].

There was not a clear seasonal effect and considerable differences in the studied antibiotic resistance genes occurrence and affluence in raw, treated and untreated sewage samples and phage and bacterial DNA fractions analysis of different seasons showed no significant relation with different prevalence of antimicrobial resistance genes in the wastewater. Spring, summer and autumn had the same rate of abundance of antibiotic resistance genes while this rate was a bit lower in winter. When looking at phages, no obvious tendency could be found in wastewater, and the highest prevalence of antibiotic resistance genes in phages was within summer while the lowest abundance was observed in spring. Although, our data analysis showed no significant correlation in this regard, some other studies have reported a noticeable seasonal effect and remarkable differences regarding the prevalence of antibiotic resistance genes in water and sediment samples but there is not any upstanding statement to say why all the antibiotic resistance genes did not represent similar seasonal discrepancy in phage and bacterial fractions [30, 46, 56, 57]. Electron microscopy analysis showed a great affluence of phages with contractile tails suspected *Myoviridae* (Fig. 4). The electron microscopy observation is in agreement with other researches [23, 45, 58,]. 

In summary, abundance of three clinically important antibiotic resistance genes in bacterial and phage DNA fraction purified from the urban wastewater in Shiraz, Iran, which defend the thought that phages may be a serious carrier for the horizontal transfer of antimicrobial resistance genes in the environment. The β-lactamase genes were detected in phage and bacterial DNA fractions which insulated from ambient water that is highly considerable, and the possibility of antibiotic resistance genes transmission by phages needs future explore. Also, available data is a reputable endorsement that wastewater is a hotspot for the distributing of antibiotic resistance genes in the environment [17, 48, 59, 60].

Based on our knowledge, here is the first notice of presence and abundance of *bla*_TEM_ and *bla *_SHV_ and *sul1 *genes in phage DNA fractions insulate from urban wastewater and environment in Iran. More epidemiological data in this regard bacteriophage antibiotic resistance gens are essential. In addition, the mechanisms how bacteriophages supply to transfer of antibiotic resistance genes in the aquatic environment are highly required in further researches. 
